# Supplementary material for: Sex Differences in Response to Marek’s Disease: Mapping Quantitative Trait Loci Regions (QTLRs) to the Z Chromosome
Source: Genes (Basel). 2022 Dec 21;14(1):20. doi: 10.3390/genes14010020 (PMC9859034; doi:10.3390/genes14010020)
Supplement: Supplementary file 1 [file genes-14-00020-s001.zip › genes-2094639-supplementary.pdf]

Mapping QTLRs affecting Marek's Disease resistance on chicken Chromosome Z

Supplemental

Table S1. QTLR genes.

QTLR	Gene stable ID version	Gene name	Gene description	Start	End
F6-1	ENSGALG00000033353.2	RIT2	Ras like without CAAX 2	3,471,974	3,658,288
F6-1	ENSGALG00000053587.1			3,831,101	3,888,531
F6-1	ENSGALG00000035773.2	PIK3C3	phosphatidylinositol 3-kinase catalytic subunit type 3	3,984,326	4,085,724
F6-1	ENSGALG00000046722.1			4,109,272	4,122,240
F6-1	ENSGALG00000027997.2			4,192,457	4,192,635
F6-1	ENSGALG00000050161.1			4,342,321	4,353,388
F6-1	ENSGALG00000047964.1			5,328,479	5,331,064
F6-1	ENSGALG00000050555.1			5,408,840	5,410,258
F6-1	ENSGALG00000054065.1			5,670,782	5,680,806
F6-1, P-1	ENSGALG00000038930.3			6,156,148	6,256,742
F6-1, P-1	ENSGALG00000055071.1			6,412,831	6,424,558
F6-1, P-1	ENSGALG00000046768.1			6,473,573	6,492,274
F6-1, P-1	ENSGALG00000050344.1	CELF4	CUGBP Elav-like family member 4	6,768,902	6,793,723
F6-1, P-1	ENSGALG00000002413.6			6,770,918	6,823,825
F6-1, P-1	ENSGALG00000048972.1			6,831,508	6,876,170
F6-1, P-1	ENSGALG00000002419.7	KIAA1328	KIAA1328	6,945,983	7,102,689
F6-1, P-1	ENSGALG00000026547.4	TPGS2	tubulin polyglutamylase complex subunit 2	7,102,975	7,126,925
F6-1, P-1	ENSGALG00000018534.5	AQP7	aquaporin 7	7,124,552	7,137,256
F6-1, P-1	ENSGALG00000026884.3		phospholipase A2 inhibitor and LY6/PLAUR domain containing	7,161,279	7,165,204
F6-1, P-1	ENSGALG00000002452.6	AQP3	aquaporin 3 (Gill blood group)	7,195,127	7,209,340
F6-1, P-1	ENSGALG00000030730.2	NOL6	nucleolar protein 6	7,254,427	7,288,141
F6-1, P-1	ENSGALG00000001668.7	UBE2R2	ubiquitin conjugating enzyme E2 R2	7,304,134	7,356,093
F6-1, P-1	ENSGALG00000013809.6		AD012Z	7,358,700	7,496,820
F6-1, P-1	ENSGALG00000005759.5	IFNW1	interferon omega 1	7,372,029	7,372,640
F6-1, P-1	ENSGALG00000046996.1	IFN-A	interferon type A1/A2-like	7,377,531	7,378,293
F6-1, P-1	ENSGALG00000047630.1	IFN-A	interferon type A1/A2-like	7,381,503	7,382,265
F6-1, P-1	ENSGALG00000054396.1		interferon type A1/A2-like	7,385,475	7,386,237
F6-1, P-1	ENSGALG00000053752.1	IFN-A	interferon type A1/A2-like	7,387,284	7,388,046
F6-1, P-1	ENSGALG00000054368.1		interferon type A1/A2-like	7,391,261	7,392,023
F6-1, P-1	ENSGALG00000048874.1	IFN-A	interferon	7,395,233	7,395,995
F6-1, P-1	ENSGALG00000044725.2		interferon type A1/A2-like	7,399,231	7,399,942
F6-1, P-1	ENSGALG00000050924.1	IFN-A	interferon type A1/A2-like	7,401,271	7,402,033
F6-1, P-1	ENSGALG00000033669.2			7,405,294	7,405,874
F6-1, P-1	ENSGALG00000052209.1		interferon type A1/A2-like	7,410,282	7,411,044
F6-1, P-1	ENSGALG00000053207.1		interferon type A1/A2-like	7,414,251	7,415,013
F6-1, P-1	ENSGALG00000054104.1		interferon type A3-like	7,421,956	7,422,718
F6-1, P-1	ENSGALG00000025660.2		small nucleolar RNA SNORD121A	7,438,822	7,438,906
F6-1, P-1	ENSGALG00000025641.2		small nucleolar RNA SNORD121A	7,441,643	7,441,726
F6-1, P-1	ENSGALG00000005800.6	DCAF12L2	DDB1 and CUL4 associated factor 12 like 2	7,510,336	7,540,872
F6-1, P-1	ENSGALG00000021378.6	UBAP1	ubiquitin associated protein 1	7,543,736	7,574,787
F6-1, P-1	ENSGALG00000053307.1			7,575,708	7,578,512
F6-1, P-1	ENSGALG00000005806.6	KIF24	kinesin family member 24	7,583,295	7,612,042
F6-1, P-1	ENSGALG00000027295.3	NUDT2	nudix hydrolase 2	7,613,662	7,620,056
F6-1, P-1	ENSGALG00000045636.2		uncharacterized LOC427400	7,621,689	7,637,215
F6-1, P-1	ENSGALG00000025921.3		LOC431653	7,645,535	7,647,631
F6-1, P-1	ENSGALG00000005814.6		KIAA1161	7,654,557	7,656,593
F6-1, P-1	ENSGALG00000005821.6	FAM219A	family with sequence similarity 219 member A	7,677,351	7,715,763
F6-1, P-1	ENSGALG00000005831.7	DNAI1	dynein axonemal intermediate chain 1	7,780,562	7,905,039
F6-1, P-1	ENSGALG00000030845.2	ENHO	energy homeostasis associated	7,905,544	7,906,945
F6-1, P-1	ENSGALG00000038723.2	RPP25L	ribonuclease P/MRP 25kDa subunit-like	7,909,086	7,911,236
F6-1, P-1	ENSGALG00000021365.6	DCTN3	dynactin subunit 3	7,913,360	7,921,570
F6-1, P-1	ENSGALG00000005839.6	ARID3C	AT-rich interaction domain 3C	7,926,291	7,938,483
F6-1, P-1	ENSGALG00000028996.3	SIGMAR1	sigma non-opioid intracellular receptor 1	7,940,896	7,943,905
F6-1, P-1	ENSGALG00000026195.3	GALT	galactose-1-phosphate uridylyltransferase	7,943,944	7,946,758
F6-1, P-1	ENSGALG00000028140.3	CNTFR	ciliary neurotrophic factor receptor	8,134,527	8,304,301
F6-1	ENSGALG00000005848.8	IL11RA	interleukin 11 receptor subunit alpha	8,328,565	8,349,086
F6-1	ENSGALG00000028256.3	CCL19	C-C motif chemokine ligand 19	8,363,698	8,366,694
F6-1	ENSGALG00000046192.2	CCL21	c-C motif chemokine 26-like	8,370,305	8,373,001
F6-1	ENSGALG00000050776.1			8,389,062	8,461,689
F6-1	ENSGALG00000048171.1			8,416,911	8,465,130
F6-1	ENSGALG00000045914.2			8,425,829	8,456,048
F6-1	ENSGALG00000001900.6	PHF24	PHD finger protein 24	8,501,025	8,509,540
F6-1	ENSGALG00000001918.6	DNAJB5	DnaJ heat shock protein family (Hsp40) member B5	8,525,124	8,538,233
F6-1	ENSGALG00000001986.7	VCP	valosin containing protein	8,540,925	8,562,119
F6-1	ENSGALG00000002009.5	FANCG	Fanconi anemia complementation group G	8,563,543	8,574,697
F6-1	ENSGALG00000002023.5	PIGO	phosphatidylinositol glycan anchor biosynthesis class O	8,577,948	8,591,196
F6-1	ENSGALG00000025775.2	gga-mir-6649	gga-mir-6649	8,585,251	8,585,389
F6-1	ENSGALG00000002064.7	STOML2	stomatin like 2	8,592,089	8,597,473
F6-1	ENSGALG00000002069.6	FAM214B	family with sequence similarity 214 member B	8,598,884	8,606,934
F6-1	ENSGALG00000002165.6	UNC13B	unc-13 homolog B	8,647,537	8,854,699
F6-1	ENSGALG00000002326.6		ATPase phospholipid transporting 8B4 (putative)	8,885,670	8,943,726
F6-1	ENSGALG00000002371.6	RUSC2	RUN and SH3 domain containing 2	8,966,773	9,000,212
F6-1	ENSGALG00000028411.3			9,006,673	9,015,300
F6-1	ENSGALG00000021355.5		receptor-type tyrosine-protein phosphatase S-like	9,015,759	9,024,777
F6-1	ENSGALG00000027747.2	TROJANZ	phosphotyrosine phosphatase kappa-like-Z	9,025,003	9,030,333

QTLR	Gene stable ID version	Gene name	Gene description	Start	End
F6-1	ENSGALG00000045280.2		uncharacterized LOC100858953	9,032,707	9,039,475
F6-1	ENSGALG00000027658.3	TESK1	testis-specific kinase 1	9,047,866	9,055,667
F6-1	ENSGALG00000005194.4	CD72	CD72 molecule	9,056,639	9,060,366
F6-1	ENSGALG00000021353.5		CD72 antigen-like	9,061,604	9,064,699
F6-1	ENSGALG00000002383.7	CD72AG	CD72 antigen	9,066,022	9,069,155
F6-1	ENSGALG00000029035.4		C-type lectin domain family 5, member A	9,069,756	9,071,860
F6-1	ENSGALG00000040221.2		undifferentiated embryonic cell transcription factor 1-like	9,072,456	9,075,192
F6-1	ENSGALG00000002386.6	TMEM8B	transmembrane protein 8B	9,080,563	9,084,664
F6-1	ENSGALG00000025868.3	TAF1C	TATA-box binding protein associated factor, RNA polymerase I subunit C-Z	9,087,724	9,092,161
F6-1	ENSGALG00000027418.3		histidine triad nucleotide binding protein 1	9,092,401	9,093,974
F6-1	ENSGALG00000027630.3	NPR2	natriuretic peptide receptor 2	9,096,779	9,106,881
F6-1	ENSGALG00000028118.3	MSMP	microseminoprotein, prostate associated	9,113,971	9,115,313
F6-1	ENSGALG00000002394.7	RGP1	RGP1 homolog, RAB6A GEF complex partner 1	9,116,265	9,126,065
F6-1	ENSGALG00000002412.6	GBA2	glucosylceramidase beta 2	9,126,064	9,135,875
F6-1	ENSGALG00000021848.4		avidin-like	9,136,599	9,137,711
F6-1	ENSGALG00000026655.3		avidin-related protein 2-like	9,145,291	9,146,416
F6-1	ENSGALG00000028039.3	AVR2	avidin related protein 2	9,149,010	9,150,157
F6-1	ENSGALG00000023622.4		avidin-related protein 4/5-like	9,152,592	9,154,039
F6-1	ENSGALG00000025945.3	AVD	avidin	9,164,659	9,166,088
F6-1	ENSGALG00000002523.6	CREB3	cAMP responsive element binding protein 3 like 4	9,167,587	9,172,144
F6-1	ENSGALG00000002548.7	TLN1	talin 1	9,172,857	9,204,072
F6-1	ENSGALG00000025842.4	TPM2	tropomyosin 2	9,207,127	9,219,170
F6-1	ENSGALG00000002578.7		aph-1 homolog B, gamma-secretase subunit	9,221,379	9,225,735
F6-1	ENSGALG00000021340.6	CA9	carbonic anhydrase 9	9,225,475	9,236,423
F6-1	ENSGALG00000037611.2		myosin IIIA-like	9,259,275	9,283,017
F6-1	ENSGALG00000002599.5	ARHGEF39	rho guanine nucleotide exchange factor 39	9,282,572	9,296,247
F6-1	ENSGALG00000002605.5	MRPL17	mitochondrial ribosomal protein L17	9,297,716	9,298,745
F6-1	ENSGALG00000027785.3	CCBE1	collagen and calcium binding EGF domains 1	9,303,582	9,323,467
F6-1	ENSGALG00000052480.1			9,414,544	9,419,141
F6-1	ENSGALG00000017952.2	U6	U6 spliceosomal RNA	9,439,172	9,439,274
F6-1	ENSGALG00000035325.2	PMAIP1	phorbol-12-myristate-13-acetate-induced protein 1	9,445,555	9,446,532
F6-1	ENSGALG00000048029.1			9,447,204	9,450,366
F6-1	ENSGALG00000002854.6	PDZD2	PDZ domain containing 2	9,455,347	9,647,483
F6-1	ENSGALG00000047625.1			9,589,180	9,602,788
F6-1	ENSGALG00000028267.4		golgi phosphoprotein 3	9,666,788	9,696,930
F6-1	ENSGALG00000003168.6	MTMR12	myotubularin related protein 12	9,711,679	9,742,308
F6-2	ENSGALG00000014784.6	MAST4	microtubule associated serine/threonine kinase family member 4	21,008,217	21,296,403
F6-2	ENSGALG00000023411.4	CD180	CD180 molecule	21,298,598	21,307,258
F6-2	ENSGALG00000027398.2	5S_rRNA	5S ribosomal RNA	21,328,741	21,328,853
F6-2	ENSGALG00000047586.1			21,375,203	21,406,692
F6-2	ENSGALG00000051141.1			21,410,315	21,414,779
F6-2	ENSGALG00000047121.1			21,514,091	21,529,002
F6-2	ENSGALG00000052422.1			21,553,771	21,569,265
F6-2	ENSGALG00000014786.6	PIK3R1	phosphoinositide-3-kinase regulatory subunit 1	21,600,456	21,657,260
F6-2	ENSGALG00000055083.1			21,642,595	21,642,648
F6-2	ENSGALG00000050639.1			21,696,285	21,700,466
F6-2	ENSGALG00000053270.1			21,748,962	21,756,431
F6-2	ENSGALG00000054494.1			21,843,033	21,864,499
F6-2	ENSGALG00000014787.7	SLC30A5	solute carrier family 30 member 5	21,885,739	21,906,410
F6-2	ENSGALG00000014788.5	CENPH	centromere protein H	21,910,292	21,917,147
F6-2	ENSGALG00000014789.5	MRPS36	mitochondrial ribosomal protein S36	21,916,343	21,924,487
F6-2	ENSGALG00000014790.7	CDK7	cyclin dependent kinase 7	21,927,561	21,950,303
F6-2	ENSGALG00000014798.7	SERINC5	serine incorporator 5	21,970,233	22,007,388
F6-2	ENSGALG00000014804.7	THBS4	thrombospondin 4	22,015,511	22,053,757
F6-2	ENSGALG00000014807.7	MTX3	metaxin 3	22,058,070	22,068,952
F6-2	ENSGALG00000026553.3	CMYA5	cardiomyopathy associated 5	22,126,658	22,173,992
F6-2	ENSGALG00000014810.7	TENT2	poly(A) RNA polymerase D4, non-canonical	22,175,870	22,214,446
F6-2	ENSGALG00000014813.6	HOMER1	homer scaffolding protein 1	22,241,850	22,331,531
F6-2	ENSGALG00000014819.5	JMY	junction mediating and regulatory protein, p53 cofactor	22,345,907	22,404,480
F6-2	ENSGALG00000050556.1		translation initiation factor IF-2-like	22,406,010	22,413,931
F6-2	ENSGALG00000004518.6	BHMT2	betaine--homocysteine S-methyltransferase 2	22,436,116	22,452,194
F6-2	ENSGALG00000004491.6	DMGDH	dimethylglycine dehydrogenase	22,452,215	22,497,335
F6-2	ENSGALG00000004438.6	ARSB	arylsulfatase B	22,502,339	22,568,346
F6-2	ENSGALG00000054348.1			22,582,992	22,593,599
F6-2	ENSGALG00000004437.6	LHFPL2	lipoma HMGIC fusion partner-like 2	22,593,889	22,715,686
F6-2	ENSGALG00000043918.2			22,706,176	22,714,516
F6-2	ENSGALG00000004425.7	SCAMP1	secretory carrier membrane protein 1	22,721,122	22,759,474
F6-2	ENSGALG00000004390.5	AP3B1	adaptor related protein complex 3 beta 1 subunit	22,789,928	22,948,747
F6-2	ENSGALG00000051662.1			22,902,220	22,904,751
F6-2	ENSGALG00000054660.1			22,955,085	22,959,339
F6-2	ENSGALG00000004353.6	TBCA	tubulin folding cofactor A	23,086,355	23,119,037
F6-2	ENSGALG00000026744.3	OTP	orthopedia homeobox	23,138,813	23,145,672
F6-2	ENSGALG00000004350.7	WDR41	WD repeat domain 41	23,183,991	23,205,580
F6-2	ENSGALG00000004339.6		phosphodiesterase 8B	23,210,259	23,289,222
F6-2	ENSGALG00000050330.1			23,294,173	23,294,224
F6-2	ENSGALG00000025649.2		small nucleolar RNA SNORA47	23,310,532	23,310,661
F6-2	ENSGALG00000004311.5	AGGF1	angiogenic factor with G-patch and FHA domains 1	23,312,434	23,332,349
F6-2	ENSGALG00000014994.6	CRHBP	corticotropin releasing hormone binding protein	23,367,125	23,375,581
F6-2	ENSGALG00000014989.6	S100Z	S100 calcium binding protein Z	23,387,539	23,392,421
F6-2	ENSGALG00000014984.6	F2RL1	F2R like trypsin receptor 1	23,413,354	23,422,556
F6-2	ENSGALG00000014983.6	F2R	coagulation factor II thrombin receptor	23,447,817	23,454,784
F6-2	ENSGALG00000048150.1			23,455,217	23,458,422
F6-2	ENSGALG00000014978.6	IQGAP2	IQ motif containing GTPase activating protein 2	23,461,532	23,577,608
F6-2	ENSGALG00000023379.4	F2RL2	coagulation factor II thrombin receptor like 2	23,491,652	23,495,738
F6-2	ENSGALG00000014967.6	SV2C	synaptic vesicle glycoprotein 2C	23,595,088	23,689,449
P-2	ENSGALG00000015032.6	CD274	CD274 molecule	27,663,890	27,681,451
P-2	ENSGALG00000031794.2	PDCD1LG2	programmed cell death 1 ligand 2	27,685,252	27,700,855
P-2	ENSGALG00000027292.2	gga-mir-6669	gga-mir-6669	27,711,357	27,711,464
P-2	ENSGALG00000015036.6	RIC1	RIC1 homolog, RAB6A GEF complex partner 1	27,712,512	27,762,665
P-2	ENSGALG00000000438.6	ERMP1	endoplasmic reticulum metallopeptidase 1	27,771,796	27,786,843
P-2	ENSGALG00000015045.6		monocarboxylate transporter 2-like	27,800,576	27,810,305
F6-3	ENSGALG00000005416.5	TTC39B	tetratricopeptide repeat domain 39B	31,833,333	31,861,093
F6-3	ENSGALG00000015105.6	PSIP1	PC4 and SFRS1 interacting protein 1	31,937,116	31,971,222
F6-3	ENSGALG00000052632.1			31,940,763	31,942,282
F6-3	ENSGALG00000015103.5	CCDC171	coiled-coil domain containing 171	31,978,142	32,207,290
F6-3	ENSGALG00000053886.1			32,223,128	32,226,544
F6-3	ENSGALG00000054332.1			32,263,422	32,278,000
F6-3	ENSGALG00000054261.1			32,274,970	32,281,085
F6-3	ENSGALG00000025350.2	gga-mir-1779	gga-mir-1779	32,310,284	32,310,375
F6-3	ENSGALG00000015101.6	BNC2	basonuclin 2	32,350,839	32,692,210

QTLR	Gene stable ID version	Gene name	Gene description	Start	End
F6-3	ENSGALG00000054671.1			32,745,113	32,745,143
F6-3	ENSGALG00000028298.3	CNTLN	centlein, centrosomal protein	32,755,378	32,945,968
F6-3	ENSGALG00000015096.7	SH3GL2	SH3 domain containing GRB2 like 2, endophilin A1	32,971,787	33,062,636
F6-3	ENSGALG00000020554.5	ADAMTSL1	ADAMTS like 1	33,117,991	33,546,156
F6-3	ENSGALG00000052548.1			33,153,703	33,189,587
F6-3	ENSGALG00000015071.6	FOCAD	focadhesin	34,160,694	34,260,169
F6-3	ENSGALG00000028142.3	HACD4	3-hydroxyacyl-CoA dehydratase 4	34,263,630	34,278,183
F6-3	ENSGALG00000015062.5	IFNK	interferon kappa-like 1	34,282,011	34,285,224
F6-3	ENSGALG00000051843.1		sperm-associated antigen 4 protein-like	34,293,089	34,427,349
F6-3	ENSGALG00000050028.1	gga-mir-7482-6	gga-mir-7482-2	34,300,413	34,300,469
F6-3	ENSGALG00000047600.1			34,305,315	34,336,138
F6-3	ENSGALG00000054049.1	gga-mir-7482-6	gga-mir-7482-2	34,315,494	34,315,550
F6-3	ENSGALG00000048442.1	gga-mir-7482-1	gga-mir-7482-2	34,330,880	34,330,936
F6-3	ENSGALG00000053918.1	gga-mir-7482-1	gga-mir-7482-2	34,346,218	34,346,274
F6-3	ENSGALG00000055107.1	gga-mir-7482-1	gga-mir-7482-2	34,406,920	34,406,976
F6-3	ENSGALG00000048217.1	gga-mir-7482-3	gga-mir-7482-2	34,421,954	34,422,010
F6-3	ENSGALG00000054345.1	RFKL	riboflavin kinase-like	34,447,841	34,452,877
F6-3	ENSGALG00000015107.6	PIP5K1B	phosphatidylinositol-4-phosphate 5-kinase type 1 beta	34,485,673	34,580,466
F6-3	ENSGALG00000015108.6	FXN	frataxin	34,582,882	34,592,944
F6-3	ENSGALG00000015109.6	TJP2	tight junction protein 2	34,597,386	34,662,316
F6-3	ENSGALG00000055034.1			34,597,613	34,597,652
F6-3	ENSGALG00000025512.2	gga-mir-1556	gga-mir-1556	34,656,766	34,656,851
F6-3	ENSGALG00000015110.6	FAM189A2	family with sequence similarity 189 member A2	34,698,424	34,709,646
F6-3	ENSGALG00000015111.6	APBA1	amyloid beta precursor protein binding family A member 1	34,714,860	34,792,783
F6-3	ENSGALG00000015113.6	PTAR1	protein prenyltransferase alpha subunit repeat containing 1	34,812,638	34,839,726
F6-3	ENSGALG00000015115.6	MAMDC2	MAM domain containing 2	34,893,638	34,957,995
F6-3	ENSGALG00000025424.2	gga-mir-1416	gga-mir-1416	34,934,091	34,934,179
F6-3	ENSGALG00000015118.7	SMC5	structural maintenance of chromosomes 5	34,965,606	35,020,664
F6-3	ENSGALG00000027374.3	KLF9	Kruppel like factor 9	35,032,928	35,045,290
F6-3	ENSGALG00000045505.2			35,081,630	35,086,439
F6-3	ENSGALG00000015126.6	TRPM3	transient receptor potential cation channel subfamily M member 3	35,092,121	35,350,353
F6-3	ENSGALG00000045111.2			35,181,229	35,201,251
F6-3	ENSGALG00000026673.2			35,191,414	35,191,490
F6-3	ENSGALG00000028446.2	gga-mir-204-1	gga-mir-204-1	35,192,621	35,192,724
DER	ENSGALG00000015147.7	ALDH1A1	aldehyde dehydrogenase 1 family member A1	35,905,444	35,958,334
P-3	ENSGALG00000023089.5	SSBP2	single stranded DNA binding protein 2	63,726,084	63,891,888
P-3	ENSGALG00000015616.6	ACOT12	acyl-CoA thioesterase 12	63,906,538	63,937,293
P-3	ENSGALG00000015603.6	ZCCHC9	zinc finger CCHC-type containing 9	63,940,607	63,946,893
P-3	ENSGALG00000015602.6	CKMT2	creatine kinase, mitochondrial 2	63,955,273	63,976,950
P-3	ENSGALG00000015598.6	RASGRF2	Ras protein specific guanine nucleotide releasing factor 2	63,986,799	64,109,277
P-4	ENSGALG00000008174.6	MTAP	methylthioadenosine phosphorylase	78,808,957	78,841,432
P-4	ENSGALG00000034505.2	CDKN2A	cyclin-dependent kinase inhibitor 2A (melanoma, p16, inhibits CDK4)	78,846,780	78,856,728
P-4	ENSGALG00000026137.4	CDKN2B	cyclin dependent kinase inhibitor 2B	78,858,726	78,862,135
P-4	ENSGALG00000054061.1			78,880,124	78,896,987
P-4	ENSGALG00000008188.6	TRIM36	tripartite motif containing 36	78,975,255	78,994,586
P-4	ENSGALG00000008197.6	PGGT1B	protein geranylgeranyltransferase type I subunit beta	79,004,881	79,039,363
P-4	ENSGALG00000045092.2	CCDC112	coiled-coil domain containing 112	79,040,637	79,049,732
P-4	ENSGALG00000024749.2	U2	U2 spliceosomal RNA	79,078,280	79,078,460
P-4	ENSGALG00000008204.4	FEM1C	fem-1 homolog C	79,117,516	79,130,913
P-4	ENSGALG00000008229.6	ALDH7A1	aldehyde dehydrogenase 7 family member A1	79,146,009	79,164,153
P-4	ENSGALG00000008237.6	GRAMD2B	GRAM domain containing 3	79,172,117	79,205,381
F6-4	ENSGALG00000005337.7	SNX2	sorting nexin 2	81,461,079	81,494,229
F6-4	ENSGALG00000018874.4	SNX24	sorting nexin 24	81,496,699	81,572,966
F6-4	ENSGALG00000005346.7	PPIC	peptidylprolyl isomerase C	81,573,781	81,579,815
F6-4	ENSGALG00000053199.1			81,604,134	81,609,598
F6-4	ENSGALG00000005351.6	PRDM6	PR/SET domain 6	81,605,055	81,673,923
F6-4	ENSGALG00000047872.1			81,694,546	81,705,690
F6-4	ENSGALG00000005368.6	CEP120	centrosomal protein 120	81,708,876	81,746,900
F6-4	ENSGALG00000037184.2	gga-mir-7483	gga-mir-7483	81,752,893	81,752,949
F6-4	ENSGALG00000026300.3			81,753,560	81,755,457
F6-4	ENSGALG00000054942.1			81,765,424	81,795,194
F6-4	ENSGALG00000052152.1			81,784,395	81,788,678
F6-4	ENSGALG00000023036.5			81,787,829	81,793,027
F6-4	ENSGALG00000029118.3		tRNA methyltransferase 10B	81,795,195	81,801,734
F6-4	ENSGALG00000017558.7		uncharacterized LOC768709	81,801,034	81,807,114
F6-4	ENSGALG00000005390.6	DCAF10	DDB1 and CUL4 associated factor 10	81,806,718	81,819,704
F6-4	ENSGALG00000028340.3			81,822,686	81,831,505
F6-4	ENSGALG00000047056.1			81,830,390	81,831,844
F6-4	ENSGALG00000005395.7	POLR1E	RNA polymerase I subunit E	81,832,191	81,847,651
F6-4	ENSGALG00000005354.6	ZBTB5	zinc finger and BTB domain containing 5	81,855,523	81,857,544
F6-4	ENSGALG00000005423.7	GRHPR	glyoxylate and hydroxypyruvate reductase	81,867,579	81,872,830
F6-4	ENSGALG00000028675.3	ZCCHC7	zinc finger CCHC-type containing 7	81,885,604	81,989,922
F6-4	ENSGALG00000051402.1			81,991,010	81,996,297

QTLR, QTLR serial number found by the F_6 (Table 1) and the Pools (Table 3); Start, End, bp location on the GRCg6a reference of the first and last markers in the QTLR.

Table S2. Association tests of all markers and haplotypes in the QTLRs, by location (GRCg6a).

QTLR	Marker/ Haps	Location	Distance	Line								Across Lines	Gene
				WL1	WL2	WL3	WPR1	WPR2	WL4	WL5	RIR1		
F_6-1	36053	3,605,367											RIT2
F_6-1	36305	3,630,520	25,153		5.4E-01						2.9E-01	3.8E-01	RIT2
F_6-1	40741	4,074,140	443,620		5.1E-01				8.0E-01			7.9E-01	PIK3C3
F_6-1	43029	4,302,959	228,819		5.1E-01						1.2E-01	2.5E-01	
F_6-1	46166	4,616,676	313,717		5.1E-01						9.4E-02	2.7E-01	
F_6-1	49698	4,969,878	353,202										
F_6-1	51533	5,153,327	183,449		5.1E-01				9.1E-01		9.4E-02	9.5E-01	
F_6-1	51662	5,166,291	12,964										
F_6-1	53289	5,328,932	162,641										
F_6-1, P-1	60228	6,022,814	693,882		5.2E-01						9.6E-02	2.1E-01	
F_6-1, P-1	61623	6,162,305	139,491		5.6E-01						1.1E-01	5.0E-02	CELF4
F_6-1, P-1	63820	6,382,092	219,787								8.7E-02	9.2E-01	CELF4
F_6-1, P-1	64741	6,474,175	92,083		6.5E-01							9.1E-03	CELF4
F_6-1, P-1	Haps A								9.5E-01		1.1E-01		
F_6-1, P-1	65507	6,550,716	76,541										CELF4
F_6-1, P-1	66056	6,605,675	54,959								2.3E-01	7.7E-01	CELF4
F_6-1, P-1	66834	6,683,420	77,745								1.9E-01	8.7E-01	CELF4
F_6-1, P-1	68063	6,806,390	122,970										CELF4
F_6-1, P-1	68223	6,822,355	15,965									7.5E-01	CELF4
F_6-1, P-1	69120	6,912,085	89,730										KIAA1328
F_6-1, P-1	69374	6,937,410	25,325										KIAA1328
F_6-1, P-1	70632	7,063,264	125,854		1.6E-02				1.7E-02		1.3E-01	2.7E-01	KIAA1328
F_6-1, P-1	71261	7,126,106	62,842		1.6E-02				2.3E-01		1.3E-01	8.5E-01	AQP7
F_6-1, P-1	72526	7,252,666	126,560										
F_6-1, P-1	73201	7,320,182	194,076										UBE2R2
F_6-1, P-1	73638	7,363,803	43,621										LOC407092
F_6-1, P-1	74454	7,445,437	81,634						1.5E-01			2.9E-01	LOC407092
F_6-1, P-1	76350	7,635,023	189,586						3.9E-01			1.3E-01	LOC427400
F_6-1, P-1	76508	7,650,818	15,795										
F_6-1, P-1	80013	8,001,352	350,534								8.8E-01	2.9E-01	
F_6-1, P-1	Haps B				1.6E-02						9.6E-01		
F_6-1, P-1	83027	8,302,734	301,382	1.4E-01		1.7E-01					3.9E-01	9.4E-03	
F_6-1	86041	8,604,115	301,381	1.2E-01								5.1E-01	
F_6-1	89887	8,988,761	384,646								5.5E-01	9.3E-01	RUSC2
F_6-1	89963	8,996,361	7,600								3.8E-01	6.5E-03	RUSC2
F_6-1	91271	9,127,101	130,740	1.7E-01							5.9E-01	3.0E-02	GBA2
F_6-1	93290	9,329,028	201,927		3.6E-02				6.8E-01		4.1E-01	6.0E-01	CCBE1
F_6-1	95986	9,598,684	269,656										
F_6-1	96619	9,661,956	63,272									5.5E-01	
F_6-1	96957	9,695,793	33,837								6.7E-01	4.3E-01	GOLPH3
F_6-1	97365	9,736,535	40,742	5.1E-01	7.0E-02							2.1E-01	MTMR12
F_6-1	Haps C				3.9E-02				6.8E-01				
F_6-2	21265	21,265,049	11,528,514	3.0E-01					9.7E-01			2.0E-01	MAST4
F_6-2	21626	21,626,796	361,747			4.9E-01			7.0E-01			4.4E-01	PIK3R1
F_6-2	21909	21,909,030	282,234				5.3E-02				1.5E-01	4.3E-02	
F_6-2	22236	22,236,438	327,408			4.6E-01					2.2E-01	1.2E-01	
F_6-2	22520	22,520,245	283,807			6.2E-01	5.3E-01				2.0E-01	9.7E-01	ARSB
F_6-2	Haps A			3.0E-01					9.5E-01			2.0E-01	
F_6-2	22886	22,886,932	366,687				2.5E-01					8.3E-01	AP3B1
F_6-2	22921	22,921,910	34,978			6.0E-01	4.1E-01		5.5E-01		9.4E-02	2.1E-01	AP3B1
F_6-2	23017	23,017,033	95,123				4.4E-01				8.4E-01	5.7E-01	
F_6-2	23073ta	23,017,034	1								7.4E-01	6.8E-01	
F_6-2	23073tg	23,017,035	1			6.4E-01						5.3E-01	
F_6-2	23073	23,073,692	56,657		9.6E-01	6.4E-01			2.8E-02		8.4E-01	3.2E-02	
F_6-2	23086	23,086,227	12,535		9.6E-01	6.6E-01	2.9E-01		2.8E-02		8.1E-01	3.2E-01	
F_6-2	23204	23,204,744	118,517										PDE8B
F_6-2	Haps B				9.6E-01	6.0E-01							
F_6-2	23217	23,217,357	12,613		2.7E-02	4.7E-03			5.1E-01		2.0E-01	3.2E-01	PDE8B
F_6-2	23250	23,250,769	33,412		1.4E-01	2.4E-01	9.5E-01		5.1E-01		2.8E-01	4.2E-01	PDE8B
F_6-2	23277	23,277,739	26,970		1.4E-01	2.3E-01	9.5E-01		4.9E-01		1.8E-01	9.6E-01	PDE8B
F_6-2	23556	23,556,398	278,659										IQGAP2
F_6-2	23651	23,651,225	94,827			2.6E-01			1.6E-03			5.3E-01	SV2C
F_6-2	Haps C						9.4E-01				2.8E-01	5.8E-01	
P-2	26696	26,696,538	3,045,313		7.9E-01							6.2E-03	RFX3
P-2	26719	26,719,600	23,062		7.9E-01							6.2E-03	RFX3
P-2	26906	26,906,396	186,796										GLIS3
P-2	26939	26,939,448	33,052										GLIS3
P-2	27025	27,025,509	86,061										
P-2	27034	27,034,536	9,027										

QTLR	Marker/ Haps	Location	Distance	Line								Across Lines	Gene
				WL1	WL2	WL3	WPR1	WPR2	WL4	WL5	RIR1		
P-2	27580	27,580,124	545,588				2.4E-01	1.9E-01				4.3E-01	JAK2
P-2	27710	27,710,563	130,439		7.3E-01		2.5E-01	2.6E-01				4.4E-01	LOC112530672
P-2	27849	27,849,293	138,730				2.0E-01					2.3E-01	KIAA2026
P-2	28046	28,046,457	197,164		8.1E-02		2.0E-01	3.0E-01				1.6E-03	GLDC
P-2	28221	28,221,317	174,860				4.1E-01	3.5E-01				2.7E-01	KDM4C
P-2	Haps											2.3E-05	
F_6-3	31832	31,832,156	3,610,839		7.6E-01	4.6E-01	1.6E-01	3.4E-01	4.3E-02			9.1E-01	
F_6-3	32118	32,118,038	285,882		7.7E-01	6.9E-01			6.5E-02			3.4E-01	CCDC171
F_6-3	32381	32,381,617	263,579				5.6E-02	9.5E-02				2.3E-01	BNC2
F_6-3	32731	32,731,634	350,017			1.2E-01			4.4E-01			7.7E-02	
F_6-3	33216	33,216,121	484,487										ADAMTSL1
F_6-3	33424	33,424,790	208,669										ADAMTSL1
F_6-3	33582	33,582,800	158,010										
F_6-3	Haps A								4.7E-01			6.1E-01	
F_6-3	33661	33,661,217	78,417					4.4E-01				1.1E-01	
F_6-3	33912	33,912,191	250,974										
F_6-3	33962	33,962,980	50,789					3.2E-01				1.3E-01	
F_6-3	34179	34,179,633	216,653					9.8E-01				3.2E-01	FOCAD
F_6-3	34513	34,513,217	333,584										
F_6-3	34522	34,522,531	9,314									8.1E-01	
F_6-3	34789	34,789,565	267,034			5.6E-01		6.5E-01	2.9E-01			3.4E-01	
F_6-3	35054	35,054,652	265,087		5.3E-01	7.5E-01		9.8E-01				9.9E-01	
F_6-3	35113	35,113,845	59,193		5.8E-01	6.7E-01						9.6E-01	TRPM3
F_6-3	35323	35,323,399	209,554		4.6E-01							1.7E-01	TRPM3
F_6-3	35383	35,383,364	59,965									6.7E-01	
F_6-3	Haps B								2.9E-01			9.8E-01	
P-3	632931	63,293,165	27,002,440	8.7E-01			7.5E-01		6.7E-01			9.3E-01	
P-3	636773	63,677,398	384,233	1.1E-01			4.6E-01	4.6E-02				5.8E-01	
P-3	637662	63,766,213	88,815						6.6E-01			7.8E-01	SSBP2
P-3	637932	63,793,285	27,072										SSBP2
P-3	639060	63,906,067	112,782				4.0E-01	4.7E-02				9.7E-01	
P-3	639115	63,911,574	5,507	8.5E-01			5.0E-01	4.7E-02	6.5E-01			7.9E-01	ACOT12
P-3	640300	64,030,006	118,432										RASGRF2
P-3	640855	64,085,517	55,511	9.9E-01					7.0E-01			5.9E-01	RASGRF2
P-3	641390	64,139,066	53,549	9.7E-01					8.4E-01			7.4E-01	
P-3	642992	64,299,292	160,226										FAM151B
P-3	645169	64,516,969	217,677										
P-3	645835	64,583,579	66,610	9.5E-01			9.9E-01		8.4E-01			4.0E-01	
P-3	Haps											4.4E-01	
P-4	73225	73,225,511	8,641,932				2.9E-02	6.3E-02	4.9E-01			4.0E-01	GTF2H2
P-4	73435	73,435,755	210,244				3.6E-02	2.9E-01	7.4E-01			9.8E-01	
P-4	78892	78,892,243	5,456,488				1.0E-08	1.7E-04				6.1E-12	
P-4	79212	79,212,166	319,923		9.9E-01		6.8E-09	8.7E-05		6.2E-01	1.4E-01	2.6E-07	
P-4	79463	79,463,296	251,130				7.2E-09	1.7E-04				4.2E-12	
P-4	79671	79,671,839	208,543				9.6E-09	1.7E-04				1.3E-13	
P-4	79878	79,878,110	206,271	1.5E-02	9.2E-01		2.4E-02	1.5E-03				4.4E-01	
P-4	Haps								7.2E-01	6.3E-01	4.6E-02	1.9E-03	
F_6-4	81514	81,514,684	1,636,574					4.1E-01			1.3E-01	5.1E-01	
F_6-4	81713	81,713,492	198,808		1.7E-02		1.5E-02	1.0E-01	2.3E-01		4.5E-01	2.5E-02	
F_6-4	81750	81,750,092	36,600		1.6E-02		5.8E-02	1.9E-01	3.3E-01			3.5E-01	
F_6-4	81795	81,795,629	45,537				1.6E-01	5.8E-01				8.8E-01	
F_6-4	81840	81,840,502	44,873	7.7E-01	2.8E-01		5.1E-01	6.0E-02	7.6E-01			7.9E-01	
F_6-4	81892	81,892,924	52,422	7.3E-01	2.8E-01		5.8E-01	3.5E-02				8.4E-01	
F_6-4	82053	82,053,490	160,566				7.2E-01					7.8E-01	
F_6-4	82142	82,142,145	88,655										
F_6-4	Haps										4.1E-01		

QTLR: QTLR serial number found by the F_6 (Table 1) and the Pools (Table 3); Marker/Haps, marker or haplotypes tested, where Haps are the haplotypes of all markers preceding this Haps and are downstream the previous Haps (for example, Haps B in the F_6 + P-1 regions, includes Markers 65507 - 80013); bp, location on GGZ (haplotypes have no specific location); Distance,

bp between markers; Line, test within a line; Across lines, test across all lines; pink highlight, $P \leq 0.05$; Gene, a gene found in the QTLR (Table S1).

Table S3. Distribution of P values in the QTLRs.

P	Markers + Haps		%	
	Lines	Ac Lines	Lines	Ac Lines
≤ 0.1	50	18	0.236	0.180
>0.1 - ≤0.2	26	9	0.123	0.090
>0.2 - ≤0.3	25	12	0.118	0.120
>0.3 - ≤0.4	7	8	0.033	0.080
>0.4 - ≤0.5	21	11	0.099	0.110
>0.5 - ≤0.6	22	9	0.104	0.090
>0.6 - ≤0.7	18	6	0.085	0.060
>0.7 - ≤0.8	15	9	0.071	0.090
>0.8 - ≤0.9	9	6	0.042	0.060
>0.9 - ≤1.0	19	12	0.090	0.120
Sum	212	100	1.000	1.000

Markers + Haps, P values of the markers and haplotypes combined; %, proportions; Lines, tests within lines; Ac Lines, tests across lines.

Table S4. File Table 'S4 - QTLRs LD.pdf'. LD matrices found in seven of the eight pure lines by individual genotyping of QTLR genes (there was only one informative marker in Line WL5). Gene, QTLR gene (Tables S1 and S2); bp, location on GGZ; Dis., bp between markers; QTLR, QTLR serial number found by the F_6 and the Pools (Tables 1 and 3); Marker, marker tested; LD values: red, $r^2 \geq 0.7$; pink, $0.15 \geq r^2 < 0.7$; white, $r^2 < 0.15$; P, P-value of the Trend association test (Table S2): pink, $P \leq 0.05$; white, $P > 0.05$. Different LD blocks have different colors.

Table S4 - QTLRs LD.pdf

Details of QTLR association and LD analyses

Thorough examination of the distribution of the P-value locations and LD pattern, revealed some interesting observations. Three of the QTLRs are presented the main text. The rest are detailed below.

1.1. QTLR P-2

None of the 13 tests within lines was significant (Table S2), while three markers and the haplotypes (Haps) were highly significant in the Across Line tests (markers P = 6.2-1.6E-3; Haps P = 2.3E-5). The first two significant Across Lines markers (Markers 26696 and 26719) were informative only in Line WL2, where they were not significant. The downstream significant Marker 28046 was informative in three lines, where it approached significance in Line WL2, but not in Lines WPR1 or WPR2.

Such results suggest two possible causative elements distributing in some of the lines, which were not informative in the markers tested. One causative element located in the region of the *RFX3* gene (Regulatory Factor X3), which influences HLA Class II expression [48]. The second putative element located in the *GLDC* gene (Glycine Decarboxylase), which is located in a differential expressed region upon MD challenge identified by Chu et al. [49]. *GLDC* is involved in human encephalopathy [50] and neural tube defects [51].

1.2. QTLR F6-3

QTLR F_6-3 was confirmed only in Line WL4 by a single marginal significant test and one test approaching significance (Table S2). Hence, taking a conservative approach, this confirmation of F_6-3 should be taken with caution.

1.3. QTLR P-3

Unfortunately, Line WL2, where QTLR P-3 was identified, was found to have no informative markers in the examination of this QTLR (Table S2). All three markers tested in

Line WPR2 were significant with very similar P-values. No other tested line was significant. The three significant markers in Line WPR2 formed a high LD block (Table S4), suggesting a single causative element. The first two markers are on either side of the *SSBP2* gene, and the third one is in the *ACOT12* gene. The putative tumour suppressor SSBP2 (Single Stranded DNA Binding Protein 2) has been linked with glioblastoma survival [52] and ACOT12 (Acyl-CoA Thioesterase 12) suppresses metastases [53] and has been linked to leukoencephalopathy [54] in human.

1.4. QTLR F6-4

Five significant tests were obtained in this QTLR (Table S2). Markers were significant in Lines WL2, WPR1 and Across Lines. Marker 81713 was significant in Lines WL2 and WPR1, and in the Across Lines test. The next marker, 81750, was significant in Line WL2 only, but approached significance in Line WPR1 (P = 5.8E-02). Another downstream marker, 81892, was significant only in Line WPR2. The two significant markers 81713 and 81750 were in the same high LD block in Line WL2 (Table S4), suggesting a single causative element distributing in this line. They did not have appreciable LD in Line WPR1, suggesting Marker 81713 - the one significant in three tests - to be closest to that causative element.
